# Identification and Contribution of Inflammation-Induced Novel MicroRNA in the Pathogenesis of Systemic Lupus Erythematosus

**DOI:** 10.3389/fimmu.2022.848149

**Published:** 2022-04-04

**Authors:** Ram P. Singh, Bevra H. Hahn, David S. Bischoff

**Affiliations:** ^1^Research Service, Veteran Administration Greater Los Angeles Healthcare System, Los Angeles, CA, United States; ^2^Division of Rheumatology, University of California, Los Angeles, Los Angeles, CA, United States; ^3^Department of Medicine, University of California, Los Angeles, Los Angeles, CA, United States

**Keywords:** microRNAs, estradiol, systemic lupus erythematosus, cytokines/chemokines, inflammation

## Abstract

Recently microRNAs (miRNAs) have been recognized as powerful regulators of many genes and pathways involved in the pathogenesis of inflammatory diseases including Systemic Lupus Erythematosus (SLE). SLE is an autoimmune disease characterized by production of various autoantibodies, inflammatory immune cells, and dysregulation of epigenetic changes. Several candidate miRNAs regulating inflammation and autoimmunity in SLE are described. In this study, we found significant increases in the expression of miR21, miR25, and miR186 in peripheral blood mononuclear cells (PBMCs) of SLE patients compared to healthy controls. However, miR146a was significantly decreased in SLE patients compared to healthy controls and was negatively correlated with plasma estradiol levels and with SLE disease activity scores (SLEDAI). We also found that protein levels of IL-12 and IL-21 were significantly increased in SLE patients as compared to healthy controls. Further, our data shows that protein levels of IL-12 were positively correlated with miR21 expression and protein levels of IL-21 positively correlated with miR25 and miR186 expression in SLE patients. In addition, we found that levels of miR21, miR25, and miR186 positively correlated with SLEDAI and miR146a was negatively correlated in SLE patients. Thus, our data shows a dynamic interplay between disease pathogenesis and miRNA expression. This study has translational potential and may identify novel therapeutic targets in patients with SLE.

## Introduction

Systemic lupus erythematosus (SLE) is an autoimmune disease associated with pathogenic autoantibodies. Genetic predisposition and epigenetic changes/regulation play a significant role in disease pathogenesis in addition to a host of other factors (e.g., gender, hormonal, environmental). Failure to maintain immune tolerance and epigenetic homeostasis may lead to aberrant and/or dysregulated gene expression that can result in loss of immune tolerance, inflammation and development of systemic autoimmunity.

Recent studies have provided further evidence that microRNAs contribute to inflammation in lupus pathogenesis (1–5). MicroRNAs (miRNAs) are small single-stranded (18-25 nucleotides), non-protein-coding RNA molecules that modulate gene expression at the post-transcriptional level. Indeed, recently, miRNAs have emerged as powerful regulators of many genes and pathways involved in the pathogenesis of inflammatory diseases (6–12). The precise role and targets of miRNAs, including their influence on disease pathogenesis, are poorly understood. Dysregulated expression of miRNAs has been reported in SLE patients (10, 13–16). Under-expression of certain miRNAs has been shown to be negatively correlated with SLE disease activity (SLEDAI) and IFN scores (14, 17, 18).

In this study, specific miRNAs were chosen based on literature, their expression pattern, and probable role in lupus pathogenesis. Since miRNAs are involved in many biological functions including inflammation and abnormal expression of some miRNAs is associated with SLE, we examined whether miR21, miR25, miR146a, and miR186 expression profiles in SLE patients’ PBMCs are different from those in healthy controls. The aim of the manuscript is to determine specific miRNAs role and relationship with estradiol, pro-inflammatory cytokines, and disease activity in SLE patients. Recent evidence also suggests that estrogens may contribute to gender bias in SLE by modulating selected miRNAs expression (19–21). Thus, miRNAs play important roles in the pathogenesis of SLE, and estrogens may regulate these miRNAs and their functions. However, an intricate balance/interaction between miRNAs and disease pathogenesis and SLE disease activity are beginning to be explored and not yet completely clear. Moreover, how pro-inflammatory cytokines and miRNAs are interconnected is not fully understood. In this translational study, we provide novel insights regarding candidate miRNAs and their interaction and mechanistic correlation with sex hormones (17β-estradiol), pro-inflammatory cytokines, and SLEDAI in SLE patients, and compare the data with healthy controls. Our data shows a dynamic interplay between SLE disease pathogenesis and miRNA expression and pro-inflammatory cytokines.

## Materials and Methods

### Subjects

We enrolled 20 subjects who were 23 years or older (23-66 years of age) and fulfilled the American College of Rheumatology revised criteria for the classification of SLE (22, 23) and 8 healthy donors with no history of autoimmune disease. Subjects’ characteristics, including age, sex, clinical parameters, medications and SLEDAI score, are shown in **Table 1**. Patients’ inclusion and exclusion criteria were described earlier (24, 25). In brief, only patients with stable disease activity [SLEDAI <6 and not >6 for the past 2 visits using immunosuppressive drugs, such as glucocorticoids and mycophenolate (1-2 g/day), at stable doses for the past two months, and daily prednisone doses not to exceed 10-15 mg/day] were recruited for the study. SLE disease activity index (SLEDAI) was recorded as described (26). The study was approved by the Institutional Review Board of the University of California Los Angeles. Written informed consent was obtained from each subject who participated in the study.

**Table 1:** Healthy Controls and SLE Patient Demographics, Clinical Parameters, Medications and Disease Characteristics Including SLEDAI Score.

Subjects	Mean Age (SD)	Gender (%)	Ethnicity (%)	ESR (SD)	CRP (SD)	ANA	Anti-dsDNA Ab (SD)	SLEDAI (SD)	Medications
**SLE**	38 (13.54)	Female (75%) Male (25%)	Asian (10%) Hispanic (25%) White (65%)	25.77 (26.1)	10.60 (8.62)	11/20 +ve	429.28 (188.33)	6.26 (4.96)	Prednisone, hydroxychloroquine, methotrexate, plaquenil, cellcept, protonix, calcium, Vitamin C, Imuran, folic acid, Vitamin D, Topomax, Colace, Atenolol, Ranitidine (mycophenolate mofetil), fish oil, furosemide
**Healthy Controls**	30 (6.13)	Female (50%) Male (50%)	Asian (25%) Hispanic (25%) White (50%)	ND	ND	ND	ND	ND	No medications

Data are presented as medians, means (SD) or number (%) as indicated. Age range was between 23-66 years, ESR (Erythrocyte sedimentation rate) range was 1-93. CRP (C-reactive protein) range was in between 0.5-16.4. ANA (Anti-nuclear antibody) was positive in 11 patients out of 20 patients, 4 had < 1:40 ANA. Anti-dsDNA Ab (Anti-double strand DNA Ab) range was between 202-656. SLEDAI (SLE disease activity index) range was between 1-16. ND (Not done). Medications listed are for all patients combined. Healthy controls had no medications at the time of blood draw.

### Cell Isolation and Preparation

Peripheral blood mononuclear cells (PBMCs) were isolated on a density gradient (Histopaque-1077, Sigma-Aldrich, St. Louis, MO, USA) from blood samples of SLE patients and healthy volunteers as described earlier (24, 25, 27).

### Measurement of Estradiol and Cytokines

17β-estradiol and cytokines were analyzed from the plasma of SLE patients as described earlier (24, 25). For estradiol and cytokines measurement, we obtained control and SLE plasma samples from the UCLA Rheumatology Biobank. Human IL-12 and IL-21 were measured using an ELISA kit from BioLegend (San Diego, CA, USA). Estradiol levels were measured in plasma by commercial ELISA (Calbiotech Inc., Spring Valley, CA) as per manufacturer’s instructions.

### RNA Isolation, miRNA Expression, and Real-Time PCR Analyses

RNA was isolated from PBMCs with TRIzol (Invitrogen Inc., Carlsbad, CA). Candidate miRNAs were analyzed using real-time PCR performed as described earlier (28–32). For miRNA analyses, total RNA was first converted to cDNA using SuperScript™ III First-Strand Synthesis System (Invitrogen) using oligo-dT primer. PCR was then performed with converted cDNA as per manufacturer’s protocol using TaqMan technology on an ABI Prism 7900 HT Sequence Detection System (Applied Biosystems, Foster City, CA, USA). For quantitation, a standard curve was constructed for each primer and probe set. All of the samples were run in either duplicate or triplicate. miR21, miR25, miR146a, miR186, and RNU48 (small nucleolar control RNA) primers and probes were obtained from Applied Biosystems (Foster City, CA, USA). Values were normalized to RNU48.

### Statistical Analyses

Data was analyzed using Prism 4.0 (GraphPad Software, San Diego, CA). Comparisons between two groups were performed using unpaired one- or two-tailed Student’s *t* test. A Shapiro-Wilk normality test was performed to confirm normality of the data, and if the data did not pass the Shapiro-Wilk test, a two-tailed Mann-Whitney test was applied for data analyses. Linear regression analysis (Spearman/Pearson) was performed to correlate miRNAs, SLEDAI, 17β-estradiol levels, IL-12p40 expression levels, and IL-17 or IL-21 protein levels. Results are expressed as mean ± SEM. p<0.05 was considered significant.

## Results

### Pro-Inflammatory miRNAs, miR21, miR-25, and miR-186 Were Significantly Increased in Lupus Patients’ PBMCs Compared to Healthy Controls; Anti-Inflammatory miR146a Was Significantly Decreased in SLE Patients Compared to Healthy Controls

**Figure 1:**
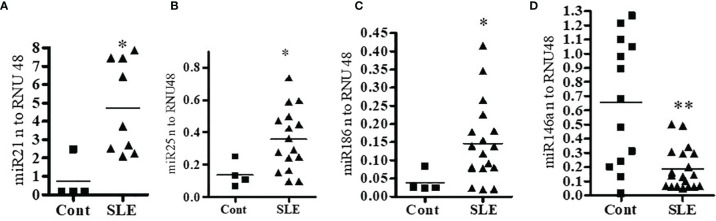
Pro-inflammatory miRNAs (miR21, miR25 and miR186) were significantly increased in lupus patients’ PBMCs compared to healthy controls. Anti-inflammatory miR146a was significantly decreased in SLE patients compared to healthy controls. Peripheral blood mononuclear cells (PBMC) (1-2 x10^6^ cells) were isolated from healthy controls (n=4-13) and SLE patients (n=8-20) and RNA was isolated. 100 ng of RNA was used for cDNA synthesis and for real-time PCR analysis with specific primer and probes of **(A)** miR21, **(B)** miR25, **(C)** miR186, and **(D)** miR146a. Primers and probes were obtained from Applied Biosystems. PCR reactions were performed on an ABI Prism 7900 HT Sequence Detection System (Applied Biosystems, Foster City, CA, USA) using TaqMan technology. All samples were run in duplicates. All values were normalized to those of RNU48 levels. Mann-Whitney two-tailed *t*-test was applied. *p < 0.05, **p < 0.001.

Since miRNAs are involved in many biological functions including inflammation and abnormal expression of some miRNAs is associated with SLE, we examined whether miR21, miR25, miR146a, and miR186 expression profiles in SLE patients’ PBMCs are different from those in healthy controls. Peripheral blood mononuclear cells (PBMC; 1-2 x10^6^ cells) from SLE patients and healthy controls were collected, cells were lysed, RNA isolated, and real-time PCR performed with specific human primers and probes (Applied Biosystems, Foster City, CA, USA). We found that SLE patients have significantly higher levels (3-6-folds) of miR21, miR25 and miR186 compared to healthy controls **(** **Figures 1A–C****)**. We also found that miR146a levels were significantly decreased in SLE patients compared to healthy controls (**Figure 1D****)**. These data suggest that SLE patients have a significantly higher pro-inflammatory miRNA signature and reduced anti-inflammatory miRNAs compared to healthy controls.

### Expression of Pro-Inflammatory miRNAs Were Positively Correlated With SLE Disease Activity Index (SLEDAI) of SLE Patients; Anti-Inflammatory miR146a Is Negatively Correlated With SLEDAI Score in SLE Patients

**Figure 2:**
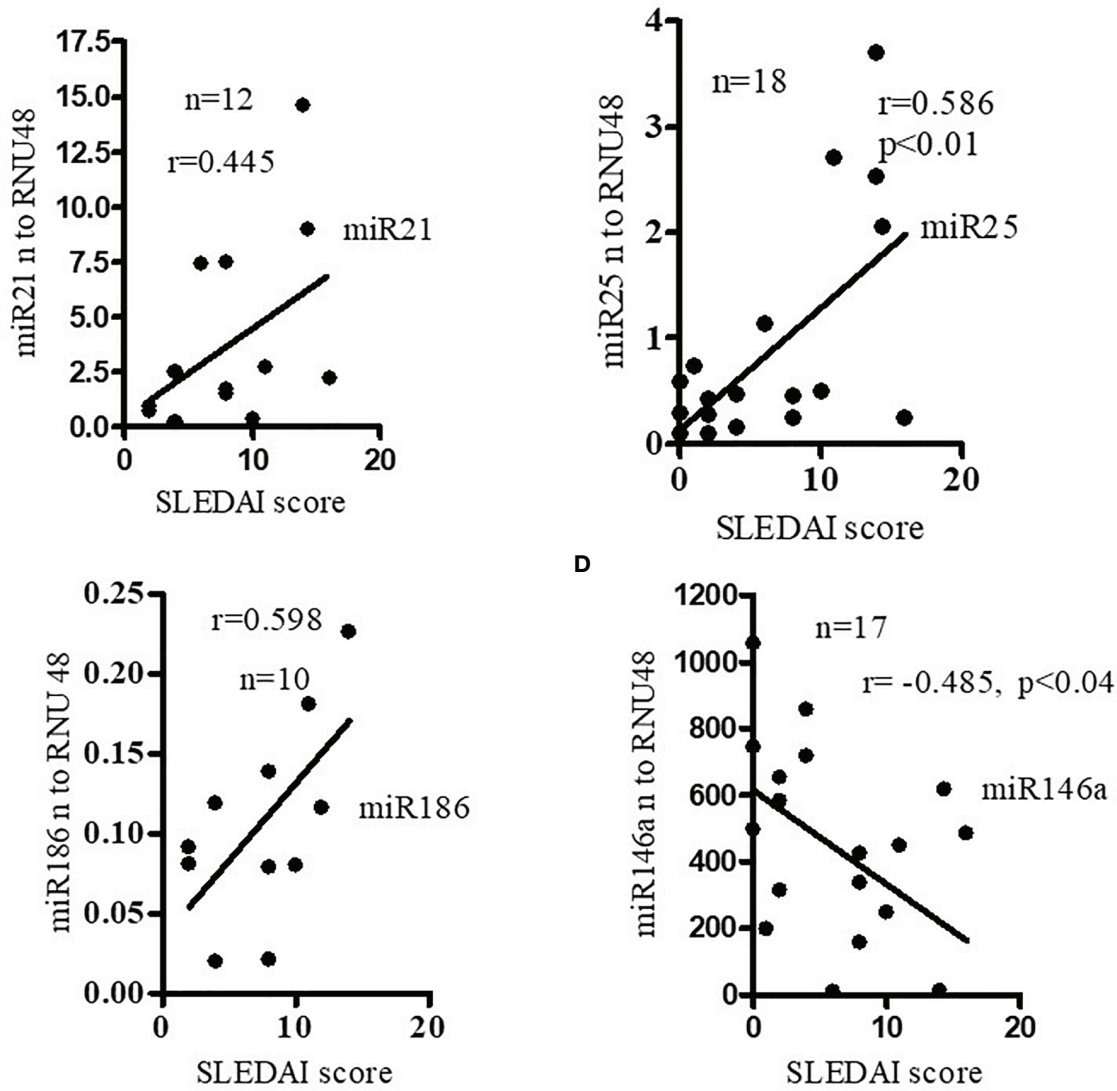
Expression levels of pro-inflammatory miRNAs were positively correlated with SLE disease activity index (SLEDAI score) of SLE patients. Anti- inflammatory miR146a levels were negatively correlated with SLEDAI score in SLE patients. Correlation of **(A)** miR21 (Spearman), **(B)** miR25 (Spearman), **(C)** miR186 (Pearson), and **(D)** miR146a (Pearson) levels and SLEDAI score in SLE patients. SLEDAI score was calculated as described previously (26). miR21 (n=12), miR25 (n=18) expression levels were determined in SLE patients with real-time PCR. miR186 (n=10), and miR146a (n=17) expression levels were correlated (Pearson) with SLEDAI score in those SLE patients. Linear regression analyses were performed between miRNAs and SLEDAI score in SLE patients.

To better understand the relationship between miRNAs, disease pathogenesis, and SLEDAI score in SLE patients, we measured SLEDAI and analyzed the candidate miRNAs in those SLE patients. We found that expression levels of pro-inflammatory miRNAs (miR21, miR25, miR186) were positively correlated with SLEDAI score **(** **Figures 2A–C****)**. In this study, we also found that miR146a levels were negatively correlated with SLEDAI **(** **Figure 2D****)**. These data clearly suggest that candidate miRNAs play a significant role in SLE pathogenesis.

### Pro-Inflammatory Cytokine (IL-12 and IL-21) Levels Were Significantly Increased in SLE Patients

**Figure 3:**
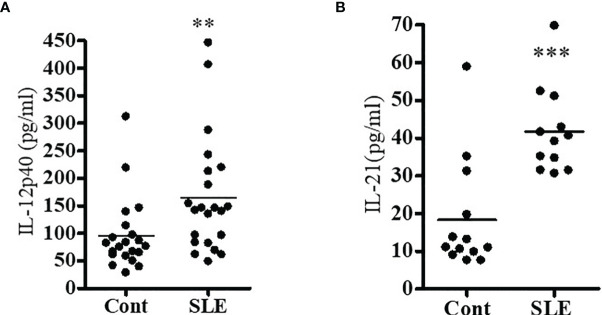
Pro-inflammatory cytokine (IL-12 and IL-21) levels were significantly increased in SLE patients. Plasma level of IL-12p40 **(A)** and IL-21 **(B)** were measured by ELISA in both healthy controls (n=16-20) and SLE patients (n=16-21). All samples were run in duplicates. A standard curve was created for each cytokine. A Shapiro-Wilk normality test was performed to confirm normality of the data, and if the data did not pass the Shapiro-Wilk test, a two-tailed Mann-Whitney test was applied for data analyses. **p < 0.001, ***p < 0.0001. Modified with ref # (25), Singh, RP, Hahn, BH and Bischoff, DS. Interferon Genes Are Influenced by 17β-Estradiol in SLE, Front Immunol. 2021; 12: 725325. doi: 10.3389/fimmu.2021.725325: Copyright: 2021 Frontiers Media SA.

Since previous clinical studies demonstrated the role of IL-12 and IL-21 in inflammation, we investigated to see whether IL-12 and IL-21 protein levels were increased in SLE patients as compared to healthy controls in our cohort. Plasma level of IL-12 and IL-21 were measured by ELISA. Indeed, our data demonstrate significantly increased protein level of IL-12 and IL-21 in SLE patients **(** **Figures 3A, B****)**. These data clearly suggest that IL-12 and IL-21 protein levels were significantly increased in SLE patients, and these cytokines play important role in lupus pathogenesis.

### Pro-Inflammatory Cytokine Levels Were Positively Correlated With miR21, miR25, and miR186 in SLE Patients

**Figure 4:**
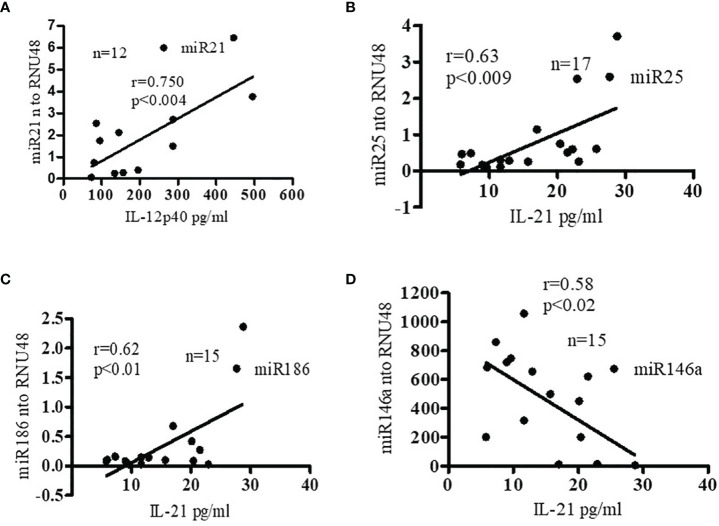
Pro-inflammatory cytokine levels were positively correlated with miR21, miR25, and miR186 in SLE patients. miR146a levels were negatively correlated with pro-inflammatory cytokine IL-21. **(A)** Correlation (Pearson) between plasma IL-12p40 protein levels and miR21 levels. Plasma IL-12p40 protein levels were measured in SLE patients (n=12) by ELISA and miR21 expression levels were determined in those patients with real-time RT-PCR. miR21 expression levels were normalized to those of RNU48 control RNA. **(B)** Correlation (Spearman) between miR25 and IL-21 levels. Plasma IL-21 protein levels were measured in SLE patients (n=17) and miR25 expression levels were determined with real-time RT-PCR. miR25 expression levels were normalized to those of RNU48 control RNA. **(C)** Correlation (Pearson) between miR186 expression levels and IL-21 protein levels in SLE patients (n=15). **(D)** Negative correlation (Pearson) between miR146a expression levels and IL-21 protein levels. (n=15). Linear regression analyses were performed between miRNAs and IL-12 or IL-21 in SLE patients. Expression levels were normalized to RNU48 RNA levels.

Recently, we reported that SLE patients have significantly increased pro-inflammatory cytokines and chemokines (25). In addition, both clinical and genetic studies have indicated roles for IL-17 (33, 34), IL-12/23 (35, 36) and IL-21 (37–40) in SLE pathogenesis. We were therefore interested to see whether pro-inflammatory cytokine protein levels of IL-12 and IL-21 correlate with candidate pro-inflammatory or anti-inflammatory miRNAs expression levels. Interestingly, we found significant positive correlations between miR21 and IL-12p40 protein levels; and with protein levels of IL-21 with miR25 and miR186 **(** **Figures 4A–C****)**. In contrast, we found a negative correlation between miR146a and IL-21 protein levels **(** **Figure 4D****)**. These data clearly indicate that pro-inflammatory cytokines and pro-inflammatory miRNAs were positively correlated and anti-inflammatory miR146a was negatively correlated in SLE patients. Thus these miRNAs play an important role in the pathogenesis of SLE. These findings are also important for prognostic and diagnostic analysis in SLE pathology. In addition, we further analyzed correlations between miR21 and IL-21, miR186 and IL-12, and miR25 and IL-12, and only found positive correlation with miR25 and IL-12 (**Supplementary Figure 1**). However, we did not address the molecular mechanisms in this study. Future detailed study will be required to pin-point the exact mechanisms of miR25 and IL-12 interactions.

### Pro-Inflammatory miRNA Expression Levels Positively Correlated With Levels of Estradiol in SLE Patients

**Figure 5:**
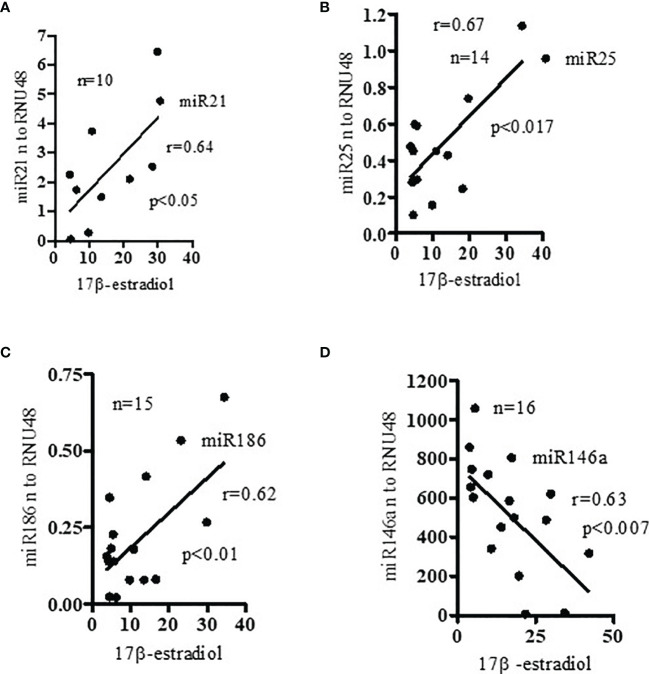
Pro-inflammatory miRNAs (miR21, miR25, miR186) levels positively correlated with levels of 17β-estradiol; and anti-inflammatory miR146a negatively correlated with 17β-estradiol in SLE patients. Plasma levels of 17β-estradiol were measured in SLE patients (n=10-15) by ELISA and miR21, miR25, miR186, and miR146a levels analyzed by real-time PCR. **(A)** miR21 (Pearson analysis), **(B)** miR25 (Pearson), and **(C)** miR186 (Spearman) expression levels were positively correlated with plasma estradiol level in SLE patients; **(D)** miR146a expression levels which were negatively correlated (Pearson). Linear regression analyses were performed between miRNAs and 17β-estradiol in SLE patients. Expression values were normalized to those of RNU48 control RNA.

Since recent evidence suggests that estrogens may contribute to gender bias in SLE by modulating selected miRNAs expression (20, 21), we measured the levels of 17β-estradiol and miRNAs in SLE patients. Our data indicate that expression levels of miR21, miR25, and miR186 **(** **Figures 5A–C**) were positively correlated with plasma 17β-estradiol levels in SLE patients. However, miR146a **(** **Figure 5D****)** expression was negatively correlated with 17β-estradiol. Earlier, we reported that 17β-estradiol levels were significantly increased in SLE patients compared to healthy controls (24). Thus, our data suggests that the levels of estradiol and expression of these miRNAs are interrelated in SLE patients, and their deep interaction may play an important role in SLE pathobiology. Future investigations will be required to address these in greater detail.

## Discussion

The present study was designed to identify, validate candidate microRNAs, and to decipher their relationship with pro-inflammatory cytokines and 17β-estradiol levels that play an important role in SLE pathology. Recent studies indicate miRNAs have a role in SLE pathogenesis (1, 5, 41). In this study, the specific miRNAs tested were chosen based on their expression and probable role in lupus pathogenesis. Both T cell and B cell-related miRNAs were described recently. B cell-related circulating miRNAs with a potential role in differential diagnosis and disease activity in lupus nephritis were described in SLE (42); in addition, miRNA-mediated control of B cell responses was also shown recently (42, 43). Several miRNAs involved in regulation of B cells, which play an important role in lupus pathogenesis, and other B cell hyperactivation and functions have been described (44–50). Similarly, T cell-related miRNAs have been described that function in inflammation and SLE (15, 51–57). We provide evidence herein that miR21, miR25, miR186 expression levels were significantly increased in SLE patients’ PBMCs compared to healthy controls. In addition, we showed that the miR146a (anti-inflammatory miRNA) expression level was significantly decreased in SLE patients **(** **Figure 1****)**. We also demonstrated a significant positive correlation between candidate miR21, miR25, miR186 and SLEDAI score in SLE patients **(** **Figure 2**). In addition, we showed that miR146a expression levels were negatively correlated with SLEDAI score **(** **Figure 2****)**. Our data agrees with other investigators that found that miR146a expression levels were significantly decreased in lupus nephritis patients compared to healthy controls (18, 58). Recently it was described that miR146a targets TRAF6 (TNF Receptor-Associated Factor 6) (18). This study postulated that miR146a reduction and TRAF6 upregulation increased the progress of ESRD (end stage renal disease). Earlier it was shown that miR146a suppressed NFκB activation and subsequent cytokine production by targeting signaling adaptor proteins, e.g., TNF receptor-associated family (TRAF)-6 and IL-1 receptor-associated kinase (IRAK)-1 (59). It was further suggested that miR146a can inhibit type 1 interferon by targeting TLR7 (Toll-like receptor-7), STAT1 (Signal transducer and activator of transcription 1), and RIG-1(Retinoic acid-inducible gene I) pathways (14, 60). In agreement with our study, the study found a reverse correlation between type 1 interferon and SLE disease activity (14). In addition, recent meta-analyses showed that miR146a expression is associated with SLE risk and there were further differences in the expression of miR146a in Asian versus Caucasian populations (61, 62). These differences in the expression level could be due to sample size, medications and active versus inactive disease status in SLE patients.

Further, we showed that protein level of IL-12 and IL-21 are positively correlated with miR21, miR25, miR186 expression levels. Our data for miR21 agreed with other investigators who have found similar increased expression level in SLE patients (13, 58, 63–69). Previously it was shown that miR21 contributes to DNA hypo-methylation in lupus CD4^+^ T cells by targeting DNA methyltransferase 1 (13). Another study provided evidence that miR21 positively regulates FoxP3 expression and negatively regulates T regulatory cell (T_reg_) development (70). Previously, we and others have demonstrated deficiency of FoxP3 expression and T_regs_ in SLE patients (24, 71). Further it was shown that silencing of miR21 *in vivo* ameliorates autoimmune splenomegaly in lupus mice (49). Thus, miR21 plays an important role in SLE disease pathogenesis.

In the current study, we investigated the expression levels of miR21, miR25, and miR186 in SLE patients and the impact on pro-inflammatory cytokines and SLE disease activity. Herein, we showed that pro-inflammatory cytokine protein levels (IL-12p40 and IL-21) were positively correlated with miR21, miR25, and miR186 expression levels **(** **Figure 4****)** and thus play an important role in SLE pathogenesis. Enhanced immune responses of PBMCs with 17β-estradiol (E2) treatment and further gene array analyses demonstrated toll-like receptor 8 (TLR8) as an E2-responsive candidate gene (72). TLR8 expression levels are up-regulated *in vivo* in SLE and in PBMCs stimulated *in vitro* with TLR8 agonists. Further, it was demonstrated that estrogen-regulated STAT1 activation promotes TLR8 expression to facilitate signaling *via* miR21 in SLE (73). It was also shown that treatment with liposomal encapsulated miR21 significantly stimulated IL-12, IL-13, and TLR8 expression and this stimulation was suppressed with MyD88 (Myeloid differentiation primary response 88) inhibition, which suggests a direct association with the TLR8-mediated signaling pathways. Further it was shown that a miR21 antagonist, chloroquine, significantly reduced TLR8 expression more than by blocking miR21 alone. Collectively, these data suggest that chloroquine may be binding to other miRNAs to prevent TLR8 induction within endosomal compartments and that multiple miRNAs are involved in TLR8 activation. Additionally, a potential role for estrogen in transcriptionally modulating many miRNAs, including miR21 has been described (74).

The potential role of miR25 and miR186 in SLE has not been explored as extensively as that of miR21. We demonstrated in this study that miR25 is upregulated in SLE patients compared to healthy controls. Similar to our study, others have used next generation sequencing and found an increased expression of miR25 in SLE patients compared to healthy controls (75). Further, it was shown that miR25 inhibits AMPD2 (adenosine monophosphate deaminase 2) in peripheral blood mononuclear cells of SLE patients (75). Over-expression of miR25 down-regulated the protein expression of AMPD2. Additionally, recent computational analysis identified the TNF-related apoptosis-inducing ligand (TRAIL) Death Receptor-4 (DR4) as a potential novel target of miR25 (76).

We also demonstrated increased expression of miR186 in SLE patients, in agreement with others (77, 78). miR186 is predicted to target major lupus susceptibility genes and is strongly associated with the predisposition to SLE disease (57, 78). In addition, it was shown that miR186 targets ROCK-1 (Rho-associated protein kinase 1) and IGF1 (Insulin-like growth factor 1) in tumor cells (79–81). Although, we were not able to study candidate miRNAs’ targets and gene ontology (GO) analyses in relation to the estradiol/hormone system (which will be investigated in greater detail in a future manuscript), we have listed candidate miRNA targets and their functions in SLE (see **Table 2**) based on current literature, and further demonstrated that four of these miRNAs are significant in inflammation and disease pathogenesis in SLE. We also demonstrated in this study that pro-inflammatory miRNA (miR21, miR25, and miR186) expression levels positively correlated with the plasma levels of 17β-estradiol in SLE patients **(** **Figure 5****)**. Previously, we showed that 17β-estradiol levels were significantly increased in SLE patients compared to healthy controls (24). Further, we showed that anti-inflammatory miR146a expression levels were negatively correlated with 17β-estradiol levels. Thus, 17β-estradiol plays a dynamic role based on candidate miRNAs’ expression levels from pro-inflammatory to anti-inflammatory pathways and vice versa. Thus, our data suggests that increased levels of pro-inflammatory miRNA together with 17β-estradiol may contribute to the female predisposition to SLE partly due to effects on SLE-susceptible genes and on pro-inflammatory pathway activation. In the present study, we also showed that 17β-estradiol, miRNAs, and pro-inflammatory cytokines are interrelated, and their deep interaction causes and influences SLE pathogenesis (**Figures 2**, **4**, **5**).

**Table 2:** MicroRNAs, their predicted targets and functions in inflammation and SLE.

miR(s)	Target(s)	Function(s)	References
miR21	RASGRP1	Activated T cell and increased proliferation, Reduced the Fas receptor- expressing B cells, Negatively regulates Treg development, regulates FoxP3 expression	(13, 49, 70)
	PDCD4		
	FoxP3		
miR25	AMPD2	Adenosine monophosphate deaminase 2 (*AMPD2*)	(75, 76)
	TRAIL-DR4	TNF-related apoptosis inducing ligand (TRAIL) death receptor-4	
miR146a	STAT1	Negative regulator of type 1 IFN pathway	(14, 60)
	IRF5		
	IRAK1		
	TRAF6		
miR186	Rho-associated protein kinase 1 (ROCK1)	Tumor suppressor, cell proliferation, migration, apoptosis, cell cycle and suppression of PI3K-Akt signaling pathway	(57, 78–81)
	IGF1 (Insulin-like growth factor 1)		

RASGRP1 (RAS guanyl nucleotide-releasing protein-1); PDCD4 (Programmed cell death protein 4); FoxP3 (forkhead box P3); AMPD2 (adenosine monophosphate deaminase 2); TRAIL-DR4 (TNF-related apoptosis inducing ligand -death receptor-4); STAT1 (Signal transducer and activator of transcription 1); IRF5 (interferon regulatory factor 5); TRAF6 (TNF Receptor Associated Factor 6); ROCK1 (Rho-associated protein kinase 1); IGF1 ( Insulin-like growth factor 1); PI3K-AKT (phosphatidylinositol 3' -kinase (PI3K-AKT); miR (microRNA).

Earlier we showed the effect of 17β-estradiol treatment on PBMCs from healthy individuals and found that 17β-estradiol significantly increased production of IL-12 (25). The role of IL-12 and the IL-23/Th17 axis has been recently demonstrated in lupus (82). Higher levels of the IL-12p40 subunit and circulating frequencies of Th17 cells were found to be correlated with SLE disease activity index (SLEDAI) including lupus nephritis (83, 84). In addition, genetic polymorphisms within the IL-12/IL12R pathways have been associated with SLE pathogenesis (85, 86). It was shown previously that several miRNAs are influenced by the estrogen levels and female sex hormone affected several lupus specific miRNA (21, 87–90). Thus, pro-inflammatory cytokines and 17β-estradiol and miRNAs are influenced by each other. The molecular interaction between miRNAs, pro-inflammatory pathways, and 17β-estradiol in SLE remains to be fully elucidated. Moreover, the molecular mechanisms by which 17β-estradiol interacts with candidate miRNAs, and pro-inflammatory cytokines in SLE are not completely clear. Future studies to delineate the detailed molecular mechanisms are required to address this interaction including target genes and inflammatory pathways.

## Data Availability Statement

The original contributions presented in the study are included in the article/**Supplementary Material**. Further inquiries can be directed to the corresponding author.

## Ethics Statement

The study was approved by the Institutional Review Board of the University of California Los Angeles. The patients/participants provided their written informed consent to participate in this study.

## Author Contributions

RPS contributed to the experimental design, obtaining funding, conducting experiments, analyzing data, preparing figures, and writing of the manuscript. BHH contributed to funding and editing of the manuscript. DSB contributed to figure and manuscript editing. All authors contributed to the article and approved the submitted version.

## Funding

This work was supported by the NIH grants AR54034, AI 083894, AI65645 to RPS; UCLA Senate Core Grant to BHH and RPS; UCLA Oppenheimer Clinical Seed Grant and American Autoimmune Related Disease Association grant to RPS.

## Conflict of Interest

The authors declare that the research was conducted in the absence of any commercial or financial relationships that could be construed as a potential conflict of interest.

## Publisher’s Note

All claims expressed in this article are solely those of the authors and do not necessarily represent those of their affiliated organizations, or those of the publisher, the editors and the reviewers. Any product that may be evaluated in this article, or claim that may be made by its manufacturer, is not guaranteed or endorsed by the publisher.
